# Drivers perceptions of road accidents: a qualitative study 

**Published:** 2019-08

**Authors:** Nader Aghakhani, Rahim Baghaei, Yousef Sheikhlou

**Affiliations:** ^*a*^Patient Safety Research Center, Urmia University of Medical Sciences, Urmia, Iran.

## Abstract

**Background:**

Road accidents is a particular public health problem that is endangering the lives of many people every year in the world. Iran is one the most dangerous countries in terms of road accidents. Human factors and driving performance are the most important causes of this car accidents. This qualitative study was conducted to determine perceptions of 12 drivers in Shiraz city about road accidents, hoping to lead to effective interventions to inform and reduce the incidence of them.

**Methods:**

12 drivers aged 24–60 years from Shiraz participated in qualitative, semi-structured, interviews about their perceptions of road accidents. The social ecological model informed interview question development, and data were examined using thematic analysis. Then the early categories formed. All interviews were recorded, categorized and analyzed.

**Results:**

The results of the study in five main themes included the sense of responsibility for driving regulations, the causes of driving hazardous behaviors, participation in modifying these behaviors, the requirements for preventive performance and the best practices for modifying behavior were emerged. Participants also believed that with more education, incentive behaviors would be more effective than punishment, accountability to their lives, occupants and people. They all opposed the increase in fines and noted that the authorities should be more concerned with measures, such as foundation of highways, the construction of parking sites, the construction of equipped centers for repair of vehicles and rest for the prevention of road accidents.

**Conclusions:**

According to the results of this study, the best and most cost-effective method is to reduce the incidence of injuries and improve the performance of drivers by using participatory and cooperative behaviors and avoiding of violence in law cases. More studies are recommended in this regard.

**Keywords:**

Qualitative research, Drivers, Road accidents

